# Fall Treatment with Fumagillin Contributes to an Overwinter Shift in *Vairimorpha* Species Prevalence in Honey Bee Colonies in Western Canada

**DOI:** 10.3390/life14030373

**Published:** 2024-03-12

**Authors:** Sarah Biganski, Oleksii Obshta, Ivanna Kozii, Roman Koziy, Michael W. Zabrodski, Midhun S. Jose, Jenna M. Thebeau, Marina C. B. Silva, Muhammad F. Raza, Fatima Masood, Sarah C. Wood, Elemir Simko

**Affiliations:** 1 Department of Veterinary Pathology, Western College of Veterinary Medicine, University of Saskatchewan, 52 Campus Drive, Saskatoon, SK S7N 5B4, Canada; oleksii.obshta@usask.ca (O.O.); midhun.jose@usask.ca (M.S.J.); jenna.thebeau@usask.ca (J.M.T.); marina.silva@usask.ca (M.C.B.S.); fahim.raza@usask.ca (M.F.R.); sarah.wood@usask.ca (S.C.W.); 2 Prairie Diagnostic Services Inc., Saskatoon, SK S7N 5B4, Canada; ivanna.kozii@usask.ca (I.K.); roman.koziy@pds.usask.ca (R.K.); michael.zabrodski@usask.ca (M.W.Z.); 3 Department of Veterinary Microbiology, Western College of Veterinary Medicine, University of Saskatchewan, 52 Campus Drive, Saskatoon, SK S7N 5B4, Canada; fatima.masood@usask.ca

**Keywords:** *Vairimorpha* spp., *Apis mellifera*, fumagillin, fall treatment, colony infection level, colony strength, survival

## Abstract

(1) Background: Microsporidiosis (nosemosis) is an intestinal disorder of adult honey bees caused by the microsporidian pathogens *Vairimorpha apis* and *Vairimorpha ceranae.* In Canada, fumagillin is an approved antibiotic used to treat this disease. However, the recommended dosage is based on efficacy studies for *V. apis*, the native pathogen in European honey bees. Since the detection of *V. ceranae* in *Apis mellifera*, *V. ceranae* became more prevalent in managed European honey bees and seems to have replaced *V. apis* due to yet unknown reasons. (2) Methods: This colony study investigated the efficacy of fumagillin administered in the fall to colonies infected with both *V. apis* and *V. ceranae* and its effects on the *Vairimorpha* species’ prevalence overwinter. Spore loads in control and fumagillin-treated colonies were analysed by microscopy; *Vairimorpha* species prevalence was determined molecularly and infection and treatment effects on colony productivity were assessed. (3) Results: Fall fumagillin treatment was associated with a temporary reduction in spore load, but there was no difference in spore loads between treated and control colonies the following spring. Interestingly, fumagillin-treated colonies had a significantly greater prevalence of *V. ceranae* relative to *V. apis* the following spring, suggesting fumagillin is less effective in controlling *V. ceranae*.

## 1. Introduction

Managed honey bees (*Apis mellifera*) play a crucial role in crop and non-crop pollination, but since 2006, an unusually high loss of colonies has repeatedly been reported in North America [1,2,3,4]. Putative contributors to colony loss are pesticides and poor nutrition, but also parasites and pathogens, and combinations of the aforementioned [3,5]. Among the concerning pathogens are two microsporidian species, *Vairimorpha* (*Nosema*) *apis* and *V*. *ceranae* (Nosematidae) [6,7,8], which are spore-forming and obligate intracellularly replicating organisms which cause the intestinal disease microsporidiosis (nosemosis) in honey bees [6,7,9,10]. This disease can be particularly devastating on an individual bee level, but is also associated with increased colony morbidity and colony mortality [11].

Since its discovery in North America in the early 2000s [12,13,14], *V. ceranae* has become the dominant *Vairimorpha* species infecting honey bee colonies, largely replacing *V. apis*, although the cause of its spread is unclear.

*V. apis* is native to the western honey bee (*Apis mellifera*), whereas *V. ceranae* was first discovered in the Asian honey bee (*Apis cerana*) [6,7,9,10]. It is unclear when exactly *V. ceranae* spread to *A. mellifera*. However, stored specimens from honey bees collected in California suggested *V. ceranae* jumped to *A. mellifera* in North America as early as 1975 and stored Canadian samples from 1994 were found positive for both *Vairimorpha* species [12,15,16]. Investigations on the widespread dispersal and prevalence of *V. ceranae* revealed it is nowadays globally distributed and more prevalent than *V. apis* [17,18]. *V. ceranae* was neither proven to be more virulent nor more infectious than *V. apis* [19,20,21] to explain its dominance in North America. Hypotheses for the success of *V. ceranae* include (i) varying host sensitivity resulting in a less effective immune response towards newly emerging pathogens [22,23,24], (ii) interspecies competition in favor of slower-replicating species [19,21,25], (iii) other concurrent colony stressors reducing honey bee resilience [26,27,28,29], (iv) or possibly decreased susceptibility of *V. ceranae* to the most commonly used antibiotic treatment for *Vairimorpha* in North America, fumagillin [30].

Fumagillin treatment for *Vairimorpha* control is recommended once colonies reach a threshold of 1 million *Vairimorpha* spores per bee and colony [31], assessed by microscopic inspection of pooled samples containing 60 bees per colony [32,33,34,35]. Fumagillin efficacy studies for the determination of the effective dosage were solely performed on *V. apis* in the past, when *V. ceranae* had not yet been discovered in western honey bees [36,37]. This dosage is still applied in commercial beekeeping today, when *V. ceranae* is more prevalent, although studies on *V. ceranae* have questioned effective control with this dosage or provided inconsistent results [30,38,39,40,41,42]. Fumagillin is an antibiotic which blocks the intracellular replication of *Vairimorpha* spp. by inhibiting the enzyme methionine aminopeptidase 2 (MetAP2), but has no effect against the resistant spores of *Vairimorpha* [11,43,44]. Studies with caged honey bees showed decreasing fumagillin concentrations (simulating the degradation of the antibiotic over time) have different effects on the suppression of spore production and the pace of resurgence of *Vairimorpha* proliferation [30].

According to reviewed colony studies on fumagillin efficacy from 1953 to 2023 [45], there have been no reports of fumagillin resistance or inefficacy in *Vairimorpha* control on the colony level in North America thus far. However, considering that the increasing *V. ceranae* prevalence suggests a replacement of *V. apis,* together with data demonstrating less effective suppression of *V. ceranae* spores during fumagillin degradation in cage bees [30], we hypothesise that fumagillin may be less effective against *V. ceranae* under field conditions as well.

## 2. Materials and Methods

To test this hypothesis, we investigated the effect of fall fumagillin treatment on *Vairimorpha* species prevalence and spore load in honey bee colonies with mixed *V. ceranae* and *V. apis* infections. Moreover, due to a lack of colony-level studies on fall fumagillin treatment efficacy against mixed *Vairimorpha* spp. infections, we compared control and fumagillin-treated colonies in terms of overwinter survival, overwinter change in colony strength, colony weight, and *Varroa* infestation, as well as overwinter colony temperature regulation.

### 2.1. Experimental Design

In May 2021, 54 nucleus honey bee colonies were produced from newly mated sister queens at the research apiary of the University of Saskatchewan (52.125858° N, −106.608381° W) in Saskatoon, Saskatchewan, Canada. In September 2021, *Vairimorpha* infection level via microscopic spore count and *Vairimorpha* species prevalence via quantitative PCR were determined for each colony. Fifty-four two-brood chamber colonies were stratified into each of the control and fumagillin treatment groups based on colony strength and *Vairimorpha* spp. infection level, with twenty-seven colonies in each control and treatment group.

On 21 September 2021, we initiated colony feeding with control or fumagillin-treated 2:1 (*w*/*v*) sucrose syrup. We administered Fumagilin-B (Vita Bee Health, Basingstoke, UK) in syrup to colonies by dissolving 10 g (active substance: 210 mg/colony, concentration 28 mg/L) in 7.5 L sucrose syrup (2:1, *w*/*v*) and top-feeding the treated syrup to colonies via inverted glass jars with a perforated lid. The labelled dosage for fall treatment of colonies with two brood chambers is 10 g Fumagilin-B dissolved in 8 L of 2:1 (*w*/*v*) sucrose syrup (active substance: 210 mg/colony, concentration 26.25 mg/L); hence, the concentration used in this experiment was slightly higher than recommended by the label. To prevent fumagillin degradation, the syrup was protected from light with an empty super. According to previous studies [40], fumagillin degrades by less than 15% within 3 weeks at temperatures below 22 °C when light-protected, similar to our feeding conditions in-field. The control group was fed with 2:1 (*w*/*v*) sucrose syrup ad libitum. The consumption of fumagillin syrup in treatment colonies was recorded during 20 days of administration and if colonies consumed 7.5 L of medicated syrup, they were given regular syrup ad libitum until they were wrapped for overwintering. All colonies were treated with amitraz (Apivar) for *Varroa* mites and oxytetracycline for metaphylaxis of American Foulbrood in fall according to label instructions, in accordance with standard commercial beekeeping practices in Saskatchewan, Canada.

### 2.2. Vairimorpha Colony Spore Load

**Figure 1:**
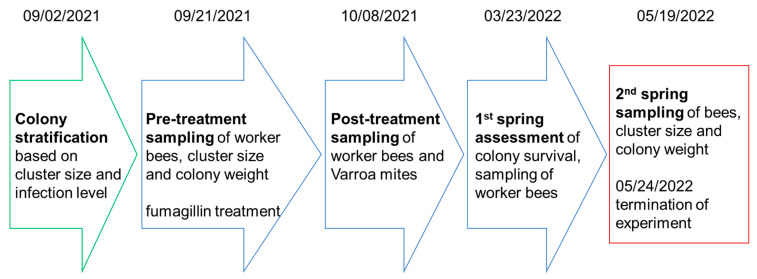
Timeline of sampling and treatment schedule of the colony study: pre-experimental phase with selection of experimental colonies (green), sampling and treatment phase (blue), and termination phase (red).

Following colony stratification on 2 September 2021, we collected 120 adult bees from each colony at 4 different times: (1) 21 September 2021 = pre-treatment, (2) 8 October 2021 = post-treatment, (3) 23 March 2022 = 1st spring assessment, and (4) 19 May 2022 = 2nd spring sampling (Figure 1). For this, we used a vacuum (Makita DCL128, Makita Canada Inc, London, ON, Canada) attached to a custom-made plastic jar [46]. Bees were collected from the periphery of the top super after opening the lid.

The 120 bees were thoroughly macerated in plastic bags with a rolling pin. After adding 120 mL of sterile water, a subsample of 1 mL was stored for spore count and the remaining homogenate was filtered through fabric mesh with approximately 0.1–0.5 mm pore size to remove larger debris from honey bee tissue. The number of spores per bee (equivalent to 1 mL) was determined in 10 μL homogenate using a hemocytometer (Neubauer, Sigma Aldrich, St. Louis, MO, USA) under light microscope (Leica, Wetzlar, Germany) [33].

### 2.3. Molecular Determination of Vairimorpha Species Prevalence

A 500 μL aliquot of the adult bee filtrate was centrifuged at 10,000× *g* for 3 min and the pellet was subjected to DNA extraction with Qiagen blood and tissue kit (Qiagen, Hilden, Germany). Each DNA sample was PCR-amplified in 20 μL reaction volumes, containing 10 μL 2× buffer (commercially premixed buffer, HotStarTaq DNA polymerase, dNTPs), 5 μL RNA-free water, both provided with the Qiagen Multiplex PCR kit (Qiagen, Toronto, ON, Canada), and 1 μL each of 10 mM primer/fluorophore-labelled probes for *V. apis* and *V. ceranae* [47], and the *Apis mellifera* housekeeping gene β-actin as internal control of the PCR [48]. According to available fluorescence filters of the thermal cycler (BioRad CFX96, C1000 Touch Thermal Cyclers, Bio-Rad Laboratories Ltd., Mississauga, ON, Canada), probes were modified as follows: *V. ceranae* probe with 5′HEX fluorophor and 3′IOWA Black FQ quencher, *V. apis* probe 5′FAM fluorophor and 3′TAMRA-SP quencher, ß-actin probe 5′Cy5 fluorophor and 3′IOWA Black RQ-Sp quencher. Multiplex quantitative PCR was performed based on the 2-step protocol obtained from to the NWP National Bee Diagnostic Centre (Beaverlodge, Canada, personal communication, adapted from [47]), with the following modified conditions in a 3-step protocol (in brackets: difference to reference): 95 °C for 5 min (10 min) initial denaturation, followed by 39 (40) cycles of 95 °C for 30 s (15 s), 57 °C for 1:30 min (60 °C, 1 min) primer annealing, and 72 °C for 30 s elongation. A 50 °C step at 2 min before initial denaturation was omitted and an elongation step that was not included in the original reference [47] was added. Primer specificity and annealing temperature for multiplex reactions containing the three primer/probe pairs were tested on positive controls containing pure *V. apis* DNA or *V. ceranae* DNA, obtained from the National Bee Diagnostic Centre, and appropriate negative controls (sterile, distilled H_2_O). The PCR conditions were validated with positive controls to exclude false-positive binding of primers on the wrong target DNA (species). Therefore, single-species DNA was tested on the tree primer/probes. The primers only amplified the respective species. Standard curves were prepared for both *Vairimorpha* species with a dilution series of purified mature spores (10 to 10^7^ spores) according to the protocol by Fries et al., 2013 [33]. Samples and standard curves were analyzed in duplicate.

### 2.4. Colony Weight and Cluster Size

All colonies were weighed pre treatment on 21 September 2021 and on 19 May 2022, using a mechanical hanging scale (Salter Model 235, Brecknell Scales, Fairmont, MN, USA) with an accuracy to the nearest 0.5 kg. To estimate colony strength, we used a 16.2-megapixel Nikon D7000 digital camera (Minato, Tokyo, Japan) with a Nikon 18 ± 105 mm lens to take a photo of an adult bee cluster on the tops and bottoms of each super early in the morning prior to bees flying out to forage. The cluster size for each super was estimated by counting and averaging the number of interframe spaces on the top and bottom of the super to the nearest 0.25 occupied by adult bees [49]. The colony cluster size was obtained by summing the cluster size for each super in the colony [50]. In March, the cluster size for the first spring assessment was based on a photo of the top super only because the second super could not be accessed due to the winter wrap. In the second spring colony assessment in May, photos of top and bottom supers were taken once colonies were unwrapped.

### 2.5. Varroa Infestation

Colony infestation with *Varroa destructor* mites in their dispersal phase was determined twice during the experiment, (1) in October 2021 after the removal of amitraz strips (Apivar) and (2) in March 2022 by collecting approx. 300 bees from a brood frame of each colony. We stored bees in winter windshield washer fluid and counted mites after shaking the samples for 30 min (alcohol wash method) to detach the mites from the bees’ bodies [51,52].

### 2.6. Temperature Monitoring

We inserted a Thermochron iButton (DS1921G-F5#, Embedded Data Systems, Lawrenceburg, KY, USA), vacuum-packed in a plastic strip, between the 5th and 6th frame of each top super on 12 October 2021, to monitor within-colony temperature hourly until 24 May 2022 (total 220 days), with a gap of 5 days between 25 March and 30 March 2022 for downloading data due to limitations of storage capacity of the iButtons. Furthermore, we randomly placed 4 iButtons in plastic strips at the experimental yard to record ambient temperature during the experiment. In-hive temperature monitoring was used to estimate the mortality date of wrapped and hence inaccessible colonies during winter.

### 2.7. Statistical Analysis

All statistical tests were conducted with R version 4.2.2 (31 October 2022) and an α = 0.05 significance level. Normality was assessed with the Shapiro–Wilk test and Levene’s test for variance homogeneity. Correlation of fumagillin consumption and spore loads at different time points was tested with non-parametric Spearman’s rank correlation test. Friedman’s two-way repeated measures ANOVA was performed for spore loads and *Vairimorpha* species prevalence, respectively, to compare data between treatments and sampling time points using pairwise comparison. The data were subjected to Bonferroni correction beforehand. Colony weight, cluster size, and *Varroa* infestation level were analyzed with two-way ANOVA for the factors time and treatment and the interaction effect of both, followed by pairwise comparison after Bonferroni adjustment. Overwinter survival was analysed by Kaplan–Meier analysis followed by log rank test.

We analyzed the temperature data from iButtons by calculating the average running daily temperature and detrended the temperature amplitude according to Meikle et al., 2016 [53]. The running daily temperature was calculated from 12 h before and after each temperature measurement. The detrended temperature was calculated by subtracting the running temperature from the temperature measurement. The daily minimum and maximum for running and detrended temperatures were calculated. Data from two control colonies were excluded from the analysis due to the similarity of in-hive temperature to ambient temperatures, which was caused by a technical defect. Temperature data were statistically analyzed by two-way ANOVA to determine the effect of time and treatment on monthly temperature differences between treatment groups, and a t-test or Mann–Whitney U test was used for pairwise comparison of average running, detrended, minimum, and maximum temperatures in colonies from the different treatment groups.

## 3. Results

### 3.1. Fumagillin Consumption

The average consumption of fumagillin syrup in the 27 treated colonies was 7.08 L (SD = 0.91 L) with a consumption rate of 459.5 mL/day (SD = 115.8 mL/day). Eleven out of twenty-seven colonies consumed the required fumagillin dosage administered in 7.5 L syrup within 14 days, ten colonies within 20 days, and six colonies consumed less than the full treatment dose within 20 days before wrapping the colonies for overwintering (average 5.63 L, SD = 1.03 L).

We found no statistical correlation between fumagillin syrup consumption and spore loads post treatment in October 2021 (S = 2651.2, *p* = 0.3407), nor in March (S = 2779.7, *p* = 0.4507) or May 2022 (S = 2940.5, *p* = 0.6112).

### 3.2. Colony Vairimorpha Infection

**Figure 2:**
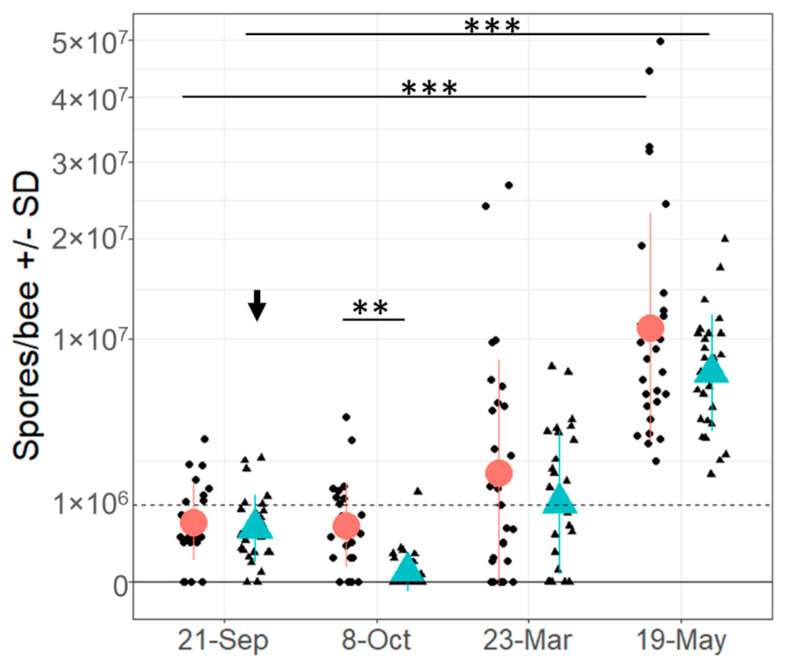
Average spore load per bee (mean ± SD) in control (red circle) and fumagillin-treated (turquois triangle) colonies from 21 September 2021 to 19 May 2022 with treatment threshold (dashed line) for fumagillin at 10^6^ spores/bee/colony. Black dots (control) and black triangles (fumagillin) indicate spore loads in individual colonies; black arrow indicates the time point of fumagillin administration. Asterisks indicate significant differences according to pairwise comparison (** *p* < 0.01, *** *p* < 0.001).

Three weeks after the initiation of fumagillin administration, treated colonies had significantly lower spore counts (10-fold) compared to control colonies (t = 3.51, df = 26, *p* = 0.002; Figure 2 and Table S1A–C for pairwise comparison). Eighteen fumagillin-treated colonies (66.6%) were *Vairimorpha* spore-free by microscopic exam post treatment in October, and six colonies had a reduced spore load compared to pre treatment in September. Hence, fumagillin decreased or eliminated *Vairimorpha* infection in 89% of the colonies. However, spore counts in March and May did not significantly differ between control and fumagillin-treated colonies (t = 1.53, df = 26, *p* = 0.139 and t = 1.99, df = 26, *p* = 0.057; Figure 2, Table S1B).

Spore load increased significantly in all colonies from September 2021 to May 2022, independent of treatment (F_(3,208)_ = 2.25, *p* = 0.084; Figure 2, but see pairwise comparison in Table S1A–C). Spore loads in May were significantly higher compared to all previous time points, but the spore loads in March were not significantly different from the pre-treatment spore loads in September in both treatments (control t = −2.53, df = 26, *p* = 0.106; fumagillin t = −2.54, df = 26, *p* = 0.106). Although there was no significant difference in spore load between untreated and treated colonies in May, the range of severity of infection was wider in untreated group; namely, 5 out of 27 colonies in the untreated control group were heavily infected (25–50 million spores per bee), whereas spore load in the fumagillin-treated group did not exceed 20 million spores per bee in any of the 27 colonies.

### 3.3. Vairimorpha *spp.* Prevalence

**Figure 3:**
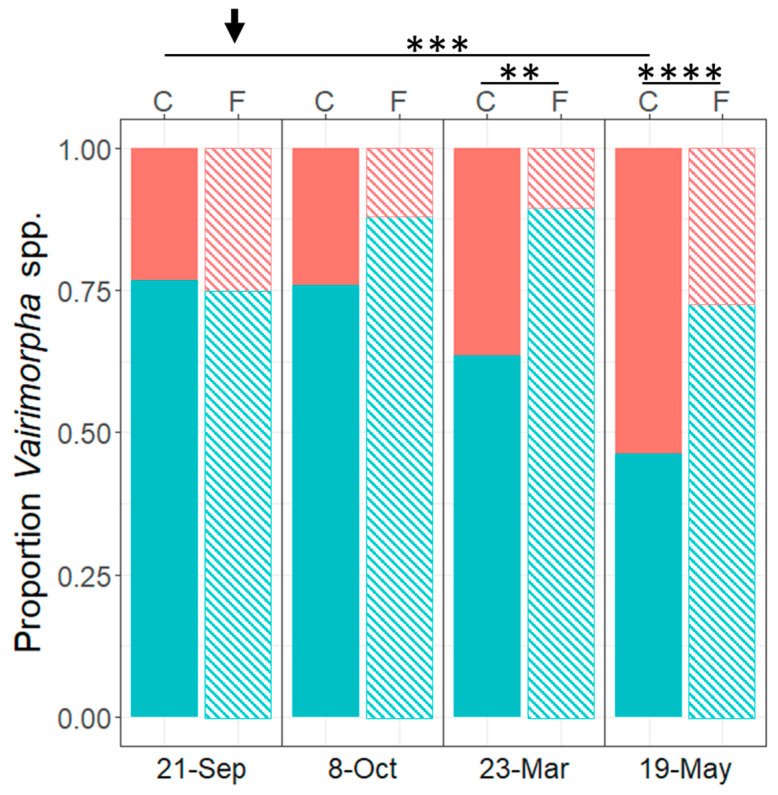
Proportion of *V. ceranae* (turquois lower bars) and *V. apis* (red upper bars) in honey bee homogenates in control (column C, filled bars) and fumagillin-treated (column F, dashed bars) colonies from 21 September 2021 to 19 May 2022. The black arrow indicates the time point of fumagillin administration (after 21 September 2021). Asterisks indicate significant differences according to pairwise comparison (** *p* < 0.01, *** *p* < 0.001, **** *p* < 0.0001).

*V. ceranae* prevalence differed significantly in both treatment groups over time and between the groups at certain time points (F_(3,208)_ = 1.230, *p* = 0.017; Figure 3, Table S2A–C). According to qPCR results, all experimental colonies had mixed infections with 75% *V. ceranae* and 25% *V. apis* before treatment on 21 September 2021 (Figure 3). Species prevalence did not significantly differ between groups in September and October 2021, but the proportion of *V. ceranae* was approximately 12% higher in October in fumagillin-treated colonies compared to controls. However, we found a significantly higher *V. ceranae* prevalence (approx. 25% higher) in fumagillin-treated colonies in March (fumagillin 89.31%, SD = 21.92 vs. control 63.48%, SD = 42.66; t = −2.79, df = 26, *p* = 0.01) and in May (fumagillin 72.1%, SD = 23.47 vs. control 46.29%, SD = 20.74; t = −4.75, df = 26, *p* < 0.001) compared to controls.

We observed a significant decrease in the proportion of *V. ceranae* from September 2021 to May 2022 in controls (t = −5.1, df = 26, *p* = 0.0002). *V. ceranae* level in the fumagillin group increased non-significantly post treatment until March 2022 and then decreased in May 2022 to the level of September 2021. Accordingly, the *V. apis* prevalence increased with the decrease of *V. ceranae*.

After fumagillin treatment in October, the majority of *Vairimorpha* spore-free (83.3%, 15 out of 18) colonies and colonies with a reduced spore count (66.6%, 4 out of 6) in the fumagillin-treated group showed a qPCR signal for *V. ceranae* but not for *V. apis*. Only two fumagillin-treated colonies were negative for both spores and *Vairimorpha* spp. DNA. The remaining treated colonies showed a *V. ceranae*/*V. apis* mix with higher proportion of *V. ceranae*.

### 3.4. Colony Strength (Cluster Size) and Weight

**Figure 4:**
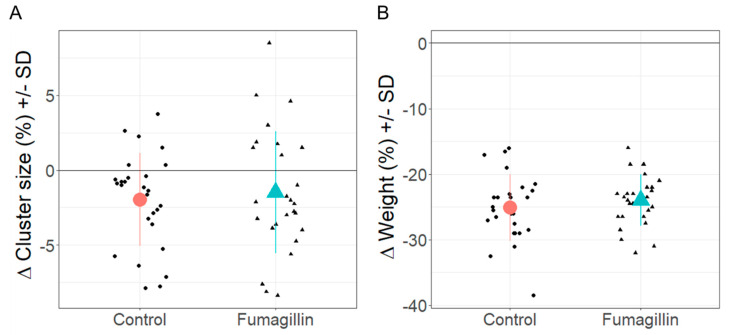
Percentage change (delta) in (**A**) cluster size (mean ± SD) and (**B**) colony weight (mean ± SD) from September 2021 to May 2022 in control (red circle) and fumagillin-treated (turquois triangle) colonies. Black dots (control) and black triangles (fumagillin) indicate individual colonies.

Both cluster size and colony weight decreased in the fumagillin-treated and control groups, but there was no significant difference between the groups for both parameters. The average cluster size per colony decreased on average by approximately two interframe spaces (−22%) in all colonies from September to May, but we only found a significant decrease in controls (control September 7.74 IFS, SD = 2.61, May 5.78, SD = 3.77; t = −3.27, df = 26, *p* = 0.003; fumagillin September 7.83 IFS, SD = 2.36, May 6.35, SD = 3.37; t = −1.89, df = 26, *p* = 0.07). However, cluster size did not significantly differ in September and May between both treatment groups (September t = −0.129, df = 26, *p* = 0.898; May t = −0.649, df = 26, *p* = 0.522; Figure 4A, displayed as change in cluster size with F_(1,52)_ = 0.243, *p* = 0.624).

Colony weight decreased significantly on average by approximately 25 kg (−35%) overwinter in all colonies, but we found no significant difference in weight change between fumagillin-treated and control colonies (control September 72.4 kg, SD = 4.93, May 47.2 kg, SD = 4.57, t = −26.1, df = 26, *p* < 0.0001; fumagillin September 68.7 kg, SD = 5.69, May 44.7 kg, SD = 5.28, t = −31.8, df = 26, *p* < 0.0001; Figure 4 B, displayed as change in weight with F_(1,51)_ = 0.834, *p* = 0.366). In September, control colonies had a slightly higher average weight (on average 3.7 kg higher) than fumagillin-treated colonies (September t = 2.71, df = 26, *p* = 0.012), but the weight did not differ in May between the treatment groups (t = 1.79, df = 26, *p* = 0.086).

### 3.5. Varroa Infestation

**Figure 5:**
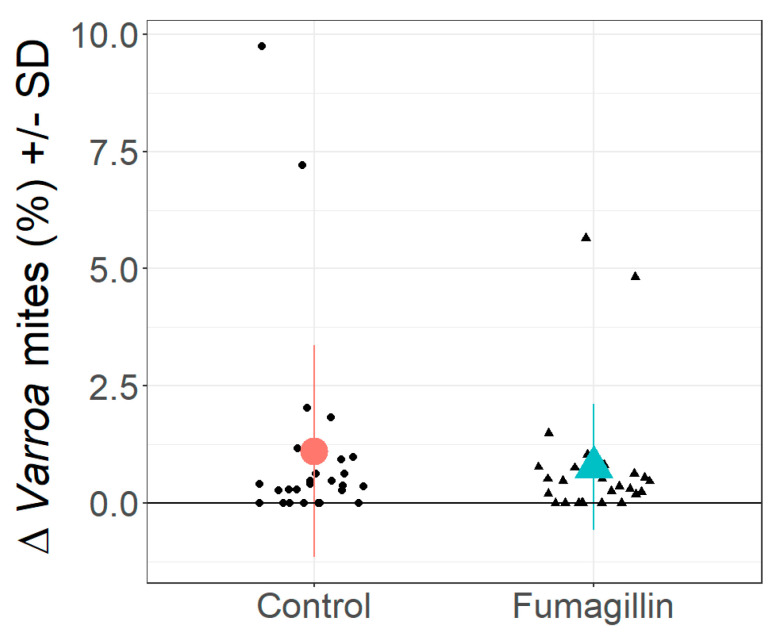
Percentage change (delta) in *Varroa* mite infestation (mean ± SD) per colony from October 2021 to March 2022 in control (red circle) and fumagillin-treated (turquois triangle) colonies. Black dots (control) and black triangles (fumagillin) indicate samples from individual colonies.

*Varroa* infestation level significantly increased overwinter in both the treatment and control groups by, on average, 0.9% (+17%) (control September 0.056%, SD = 0.205, March 1.13%, SD = 2.24, t = 2.51, df = 26, *p* = 0.019; fumagillin September 0.011%, SD = 0.055, March 0.775%, SD = 1.34, t = 2.96, df = 26, *p* = 0.007; F_(1,53)_ = 13.666, *p* = 0.0005; Figure 5, displayed as change in *Varroa* infestation with *χ*^2^ = 0.0116, df = 1, *p* = 0.914) but we found no difference in *Varroa* mite infestation between the treatment and control groups in September, nor in March (September t = 1.11, df = 26, *p* = 0.278; March t = 0.668, df = 26, *p* = 0.51).

### 3.6. Overwinter Survival and Temperature

Overwinter survival did not differ between fumagillin-treated and control colonies (Chi^2^ = 1.1, df = 1, *p* = 0.3).

One colony of the twenty-seven control colonies died between the first spring assessment in March (day 164) and the termination of the experiment in May 2022 (day 220). All 27 colonies of the fumagillin treatment group survived until the termination of the experiment.

**Figure 6:**
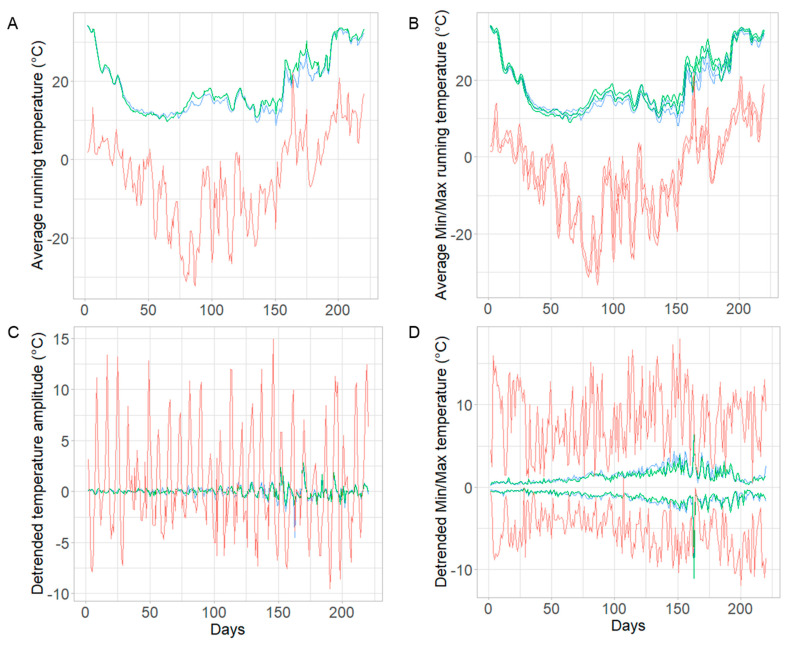
(**A**) Average daily running temperature (in °C), (**B**) minimum and maximum daily running temperature, (**C**) detrended temperature amplitude (°C), and (**D**) daily minimum and maximum of the detrended temperature amplitude of ambient temperature (red), control (green), and fumagillin-treated (blue) colonies.

In-hive temperature did not differ between treated and control colonies. We recorded the in-hive temperature to monitor overwinter colony mortality as hive inspections were not possible from November to April when colonies were wrapped due to low ambient temperatures (−40 °C) in Saskatchewan. The running monthly temperature differed significantly over time, but not between treatment and control groups (Figure 6A, time F_(7,28)_ = 62.94, *p* < 0.0001, treatment F_(1,4)_ = 0.8599, *p* = 0.4063, time*treatment F_(7,28)_ = 0.316, *p* = 0.940). The average running daily temperature did not differ among treatment and control groups (control 18.69 °C, SD = 5.16, fumagillin 19.27 °C, SD = 5.24, Figure 6A; t = 0.400, df = 49, *p* = 0.691) nor did the average daily detrended temperature (control −0.002 °C, SD = 0.004, fumagillin −0.002 °C, SD = 0.003; Figure 6C; W = 358, df = 49, *p* = 0.5435). Neither the minimum and maximum average running daily temperature (control min 17.89 °C, SD = 5.13, max 19.48 °C, SD = 5.15, fumagillin min 18.49 °C, SD = 5.32, max 20.07 °C, SD = 5.12; Figure 6B; t(min) = 0.405, df = 49, *p* = 0.6872, t(max) = 0.4096, df = 49, *p* = 0.6839) nor the minimum and maximum detrended temperature amplitude (control min −1.22 °C, SD = 0.49, max 1.44 °C, SD = 0.58, fumagillin min −1.14 °C, SD = 0.39, max 1.28 °C, SD = 0.45; Figure 6D; W(min) = 309, df = 49, *p* = 0.772, W(max) = 366, df = 49, *p* = 0.4487) differed between treatment groups.

## 4. Discussion

The colony study presented here demonstrates the influence of fumagillin fall treatment on colonies with mixed *Vairimorpha* species infection in the Canadian prairies. We found fumagillin treatment was associated with a significant reduction in *Vairimorpha* spp. spore load after treatment in fall and a significant increase in *V. ceranae* prevalence the following spring. Fumagillin treatment was not shown to have a significant effect on colony strength, weight, survival, *Varroa* infestation, or temperature homeostasis.

Fumagillin treatment in fall effectively reduced *Vairimorpha* colony spore loads short-term. With 89% efficacy, we recorded a sufficient reduction in *Vairimorpha* colony spore loads within 3 weeks of administration of fumagillin. According to microscopic spore counts, we found no spores in most of our samples in the fumagillin-treated group in October, although their spore loads increased in May (average 8 × 10^6^ spores/bee) to a similar level to that in controls, with 1.32 × 10^7^ spores/bee. We conclude the reduction in *Vairimorpha* spp. spore loads in October had no long-term effect after fall treatment, which is in strong concordance with the recently published studies on fumagillin efficacy from the US and Canada [39,54]. Both studies recorded a short-term drop in infection intensity post treatment, similar to our study. Interestingly, in our study, the range of spore loads in untreated control colonies was considerably wider than in fumagillin-treated ones, although the means were not significantly different. The clinical significance of heavy infection (25–50 million spores per bee) of ~20% of the colonies in the untreated group is unclear and should be investigated, but they could potentially be a source of infection for the adjacent colonies [35]. Contrary to our study, Prouty et al. [54] reported higher spore loads in spring in fumagillin-treated colonies than in controls. The authors discussed fumagillin causing an imbalance in the gut microbiome and hence diminishing honey bee resilience towards *Vairimorpha* infections, increasing pathogen prevalence over time in treated colonies [54,55].

We saw a significant increase in *V. ceranae* prevalence in fumagillin-treated colonies in the following spring compared to controls. Before treatment in fall, colonies in both groups had a comparable ratio of both *V. ceranae* (75%) and *V. apis* (25%). The ratio changed in the fumagillin group post treatment in favor of *V. ceranae*. In spring, we found a significant drop in *V. ceranae* in controls compared to fall. Moreover, fumagillin-treated colonies revealed a 20–25% higher *V. ceranae* level in March and May compared to controls. In concordance with other field studies [38,41], our molecular results suggest that the labelled dosage of fumagillin is more effective in the suppression of *V. apis* than of *V. ceranae* within 3 weeks after application. Cage experiments support fumagillin’s efficacy for both species when treated with the recommended concentration, which corroborates our spore count data but not our molecular data, where we still detected *V. ceranae* DNA but no *V. apis* post treatment [30]. However, the cage study demonstrated a significantly lowered efficacy in *V. ceranae* compared to *V. apis* when treated with decreasing fumagillin concentrations. While lower levels of fumagillin are still able to suppress *V. apis* spore production, the study demonstrated an increased *V. ceranae* spore proliferation after fumagillin treatment with 0.001× recommended concentration compared to untreated controls and respectively treated *V. apis* infected bees. The cage study was based on single-species infected bees, each inoculated with the same number of spores at the beginning of the experiment, whereas we used mixed-infected colonies with different initial spore levels for both *Vairimorpha* species. Hence, we focused on the relative abundance of *V. ceranae* DNA to *V. apis* DNA for species-specific efficacy estimation. We wonder if the increasing *V. ceranae* DNA level relative to *V. apis* DNA in our fumagillin-treated colonies during the spring could be explained by a comparable degradation effect described in a cage study by Huang et al. 2013 [30]. Regression models for spore counts during degradation estimated fumagillin decreases to an inefficient residue level within 2 to 5 months, coinciding with the here-presented data of the first spring assessment in March, but also with a residue analysis in Spanish colonies [30,56].

Generally, both species show seasonal proliferation patterns, with an increase in spring or early summer, respectively, and consequently compete for transmission to newly hatching nestmates [57,58]. Molecular data revealed the utter eradication of *V. apis* after fumagillin treatment in fall but *V. ceranae* DNA was still detectable at a low level, even though we did not detect spores in most colonies. Considering the proposed lower susceptibility of *V. ceranae* to fumagillin [30], remaining *V. ceranae* spores in winter can faster resume proliferation and transmission to new nestmates than less abundant *V. apis*, resulting in a higher prevalence in spring.

Despite efficient *Vairimorpha* spp. reduction in fall, we could not find strong evidence for a colony-protective or -strengthening effect of fumagillin treatment in mixed-infected colonies, which supports previous studies in Canada and Uruguay [39,41,59]. Our data showed a similar reduction in colony weight and strength (cluster size) overwinter in controls and fumagillin-treated colonies. Only two colonies in the control group had a very small remaining bee cluster (with <1 covered interframe space; one of those colonies died before the second spring assessment) in March and May 2022, although these colonies were not found to have a higher *Vairimorpha* spp. infection rate relative to colonies with larger cluster sizes. All control and fumagillin-treated colonies survived overwinter until the first spring assessment.

The weaknesses of our study include the termination of the experiment in spring. A continuation of this study into late spring/summer would have given insight into further development of species distribution and efficacy under a combined fall and spring treatment. We administered fumagillin dissolved in 7.5 L syrup (according to label instruction) over 2–3 weeks rather than applying the previously reported drench method [40] which ensures the rapid uptake of the required fumagillin dose and reduces risk for degradation [30,40].

Future directions include the continuation of such colony studies with the application of fumagillin in spring, when the experimental colonies showed infection levels above the suggested treatment threshold of 10^6^ spores/bee. Moreover, a comparative study with treatment in fall on pure *V. apis-*, *V. ceranae-*, and mixed-infected colonies with specified species ratios can elucidate the impact of fumagillin on the replacement of *V. apis* with the sister species.

## 5. Conclusions

Our colony study demonstrated that fumagillin applied in fall reduces the spore load of both *Vairimorphia* species but does not prevent significant increases in spore loads the following spring. Hence, an insufficient long-term reduction in colony infection levels would require a spring treatment to reduce spore loads in spring/summer or the implementation of an alternative IPM for *Vairimorpha* control. Furthermore, our study suggests that fall fumagillin treatment is more effective against *V. apis*, providing an advantage for *V. ceranae* proliferation in treated colonies, which could at least partially explain the increased prevalence of *V. ceranae* in the Canadian beekeeping industry.

## Data Availability

The data presented in this study are available in this article and in the Supplementary Materials [Supplementary Tables S1–S6].

## Supplementary Materials

The following supporting information can be downloaded at: https://www.mdpi.com/article/10.3390/life14030373/s1, Table S1. Friedman’s repeated-measures ANOVA statistical analysis for spore loads. Table S2. Friedman’s repeated-measures ANOVA statistical analysis for *V. ceranae* prevalence. Table S3. Raw data for cluster size, weight, *Varroa* mites, and syrup consumption. Table S4. Raw data for spore counts per colony and sampling time points. Table S5. Raw data for *Vairimorpha* spp. ratio calculated from Ct values using the slope function derived from individual-species standard curves. Slope functions are as follows: y = −3.474x − 45.741 for *V. apis* and y = −3.64x − 43.943 for *V. ceranae*. Table S6. Raw data from thermos sensors including average running temperature, average detrended temperature, and minimum and maximum running and detrended temperatures.

## References

[1] (2022). Canadian Association of Professional Apiculturists Statement on Honey Bee Wintering Losses in Canada. https://capabees.com/shared/CAPA-Statement-on-Colony-Losses-2021-2022-FV.pdf.

[2] Higes M., Martín-Hernández R., Botías C., Bailón E.G., González-Porto A.V., Barrios L., del Nozal M.J., Bernal J.L., Jiménez J.J., Palencia P.G. (2008). How Natural Infection by *Nosema ceranae* Causes Honeybee Colony Collapse: Natural *Nosema ceranae* Infection. Environ. Microbiol..

[3] Ellis J.D., Evans J.D., Pettis J. (2010). Colony Losses, Managed Colony Population Decline, and Colony Collapse Disorder in the United States. J. Apic. Res..

[4] VanEngelsdorp D., Hayes J., Underwood R.M., Pettis J. (2008). A Survey of Honey Bee Colony Losses in the US, Fall 2007 to Spring 2008. PLoS ONE.

[5] Steinhauer N., Kulhanek K., Antúnez K., Human H., Chantawannakul P., Chauzat M.-P., vanEngelsdorp D. (2018). Drivers of Colony Losses. Curr. Opin. Insect Sci..

[6] Fries I. (1993). *Nosema apis*—A Parasite in the Honey Bee Colony. Bee World.

[7] Fries I., Feng F., da Silva A., Slemenda S.B., Pieniazek N.J. (1996). *Nosema ceranae* n. pp. (Microspora, Nosematidae), Morphological and Molecular Characterization of a Microsporidian Parasite of the Asian Honey Bee *Apis cerana* (Hymenoptera, Apidae). Eur. J. Protistol..

[8] Tokarev Y.S., Huang W.-F., Solter L.F., Malysh J.M., Becnel J.J., Vossbrinck C.R. (2020). A Formal Redefinition of the Genera *Nosema* and *Vairimorpha* (Microsporidia: Nosematidae) and Reassignment of Species Based on Molecular Phylogenetics. J. Invertebr. Pathol..

[9] Kudo R. (1920). Notes on *Nosema apis* Zander. J. Parasitol..

[10] Higes M., Martín R., Meana A. (2006). *Nosema ceranae*, a New Microsporidian Parasite in Honeybees in Europe. J. Invertebr. Pathol..

[11] Botías C., Martín-Hernández R., Barrios L., Meana A., Higes M. (2013). *Nosema* spp. Infection and Its Negative Effects on Honey Bees (*Apis mellifera iberiensis*) at the Colony Level. Vet. Res..

[12] Klee J., Besana A.M., Genersch E., Gisder S., Nanetti A., Tam D.Q., Chinh T.X., Puerta F., Ruz J.M., Kryger P. (2007). Widespread Dispersal of the Microsporidian *Nosema ceranae*, an Emergent Pathogen of the Western Honey Bee, *Apis mellifera*. J. Invertebr. Pathol..

[13] Chen Y., Evans J.D., Smith I.B., Pettis J.S. (2008). *Nosema ceranae* Is a Long-Present and Wide-Spread Microsporidian Infection of the European Honey Bee (*Apis mellifera*) in the United States. J. Invertebr. Pathol..

[14] Williams G.R., Shafer A.B.A., Rogers R.E.L., Shutler D., Stewart D.T. (2008). First Detection of *Nosema ceranae*, a Microsporidian Parasite of European Honey Bees (*Apis mellifera*), in Canada and Central USA. J. Invertebr. Pathol..

[15] Traver B.E., Fell R.D. (2015). A Scientific Note: Survey for *Nosema* spp. in Preserved *Apis* spp. Apidologie.

[16] Currie R.W., Pernal S.F., Guzmán-Novoa E. (2010). Honey Bee Colony Losses in Canada. J. Apic. Res..

[17] Emsen B., Guzman-Novoa E., Hamiduzzaman M.M., Eccles L., Lacey B., Ruiz-Pérez R.A., Nasr M. (2016). Higher Prevalence and Levels of *Nosema ceranae* than *Nosema apis* Infections in Canadian Honey Bee Colonies. Parasitol. Res..

[18] Martín-Hernández R., Botías C., Bailón E.G., Martínez-Salvador A., Prieto L., Meana A., Higes M. (2012). Microsporidia Infecting *Apis mellifera*: Coexistence or Competition. Is *Nosema ceranae* Replacing *Nosema apis*?. Environ. Microbiol..

[19] Forsgren E., Fries I. (2010). Comparative Virulence of *Nosema ceranae* and *Nosema apis* in Individual European Honey Bees. Vet. Parasitol..

[20] Huang W.-F., Solter L., Aronstein K., Huang Z. (2015). Infectivity and Virulence of *Nosema ceranae* and *Nosema apis* in Commercially Available North American Honey Bees. J. Invertebr. Pathol..

[21] Milbrath M.O., van Tran T., Huang W.-F., Solter L.F., Tarpy D.R., Lawrence F., Huang Z.Y. (2015). Comparative Virulence and Competition between *Nosema apis* and *Nosema ceranae* in Honey Bees (*Apis mellifera*). J. Invertebr. Pathol..

[22] Sinpoo C., Paxton R.J., Disayathanoowat T., Krongdang S., Chantawannakul P. (2018). Impact of *Nosema ceranae* and *Nosema apis* on Individual Worker Bees of the Two Host Species (*Apis cerana* and *Apis mellifera*) and Regulation of Host Immune Response. J. Insect Physiol..

[23] Dussaubat C., Sagastume S., Gómez-Moracho T., Botías C., García-Palencia P., Martín-Hernández R., Le Conte Y., Higes M. (2013). Comparative Study of *Nosema ceranae* (Microsporidia) Isolates from Two Different Geographic Origins. Vet. Microbiol..

[24] Antúnez K., Martín-Hernández R., Prieto L., Meana A., Zunino P., Higes M. (2009). Immune Suppression in the Honey Bee (*Apis mellifera*) Following Infection by *Nosema ceranae* (Microsporidia). Environ. Microbiol..

[25] Natsopoulou M.E., McMahon D.P., Doublet V., Bryden J., Paxton R.J. (2015). Interspecific Competition in Honeybee Intracellular Gut Parasites Is Asymmetric and Favours the Spread of an Emerging Infectious Disease. Proc. R. Soc. B Biol. Sci..

[26] Pettis J.S., vanEngelsdorp D., Johnson J., Dively G. (2012). Pesticide Exposure in Honey Bees Results in Increased Levels of the Gut Pathogen *Nosema*. Naturwissenschaften.

[27] Toplak I., Jamnikar Ciglenečki U., Aronstein K., Gregorc A. (2013). Chronic Bee Paralysis Virus and *Nosema ceranae* Experimental Co-Infection of Winter Honey Bee Workers (*Apis mellifera* L.). Viruses.

[28] Guzmán-Novoa E., Eccles L., Calvete Y., Mcgowan J., Kelly P.G., Correa-Benítez A. (2010). *Varroa destructor* Is the Main Culprit for the Death and Reduced Populations of Overwintered Honey Bee (*Apis mellifera*) Colonies in Ontario, Canada. Apidologie.

[29] Holt H.L., Grozinger C.M. (2016). Approaches and Challenges to Managing *Nosema* (Microspora: Nosematidae) Parasites in Honey Bee (Hymenoptera: Apidae) Colonies. J. Econ. Entomol..

[30] Huang W.-F., Solter L.F., Yau P.M., Imai B.S. (2013). *Nosema ceranae* Escapes Fumagillin Control in Honey Bees. PLoS Pathog..

[31] (2020). Alberta Agriculture and Forestry Honey Bee Pests and Diseases—Best Management Practices. https://open.alberta.ca/publications/9781460147696.

[32] Bourgeois A.L., Rinderer T.E., Beaman L.D., Danka R.G. (2010). Genetic Detection and Quantification of *Nosema apis* and *N. ceranae* in the Honey Bee. J. Invertebr. Pathol..

[33] Fries I., Chauzat M.-P., Chen Y.-P., Doublet V., Genersch E., Gisder S., Higes M., McMahon D.P., Martín-Hernández R., Natsopoulou M. (2013). Standard Methods for *Nosema* Research. J. Apic. Res..

[34] Biganski S., Lester T., Obshta O., Jose M.S., Thebeau J.M., Masood F., Silva M.C.B., Camilli M.P., Raza M.F., Zabrodski M.W. (2023). Comparison of Individual and Pooled Sampling Methods for Estimation of *Vairimorpha* (*Nosema*) spp. Levels in Experimentally Infected Honey Bee Colonies. J. Vet. Diagn. Investig..

[35] Echazarreta J.M., Delhey V.K., Pellegrini C.N., Gallez L.M. (2023). Variability of *Vairimorpha (=Nosema) ceranae* Infection Level in Individual Honey Bees and Its Implications on the Pooled Sample Size. J. Apic. Res..

[36] Katznelson H., Jamieson C.A. (1952). Control of *Nosema* Disease of Honeybees with Fumagillin. Science.

[37] Webster T.C. (1994). Fumagillin Affects *Nosema apis* and Honey Bees (Hymonopterai Apidae). J. Econ. Entomol..

[38] McCallum R., Olmstead S., Shaw J., Glasgow K. (2020). Evaluating Efficacy of Fumagilin-B^®^ Against Nosemosis and Tracking Seasonal Trends of *Nosema* spp. in Nova Scotia Honey Bee Colonies. J. Apic. Sci..

[39] Punko R.N., Currie R.W., Nasr M.E., Hoover S.E. (2023). Effect of Fumagilin-B Treatment Timing on *Nosema* (*Vairimorpha* spp.; Microspora: Nosematidae) Abundance and Honey Bee (Hymenoptera: Apidae) Colonies under Winter Management in the Canadian Prairies. J. Econ. Entomol..

[40] Higes M., Nozal M.J., Alvaro A., Barrios L., Meana A., Martín-Hernández R., Bernal J.L., Bernal J. (2011). The Stability and Effectiveness of Fumagillin in Controlling *Nosema ceranae* (Microsporidia) Infection in Honey Bees (*Apis mellifera*) under Laboratory and Field Conditions. Apidologie.

[41] Williams G.R., Shutler D., Little C.M., Burgher-Maclellan K.L., Rogers R.E.L. (2011). The Microsporidian *Nosema ceranae*, the Antibiotic Fumagilin-B^®^, and Western Honey Bee (*Apis mellifera*) Colony Strength. Apidologie.

[42] Williams G.R., Sampson M.A., Shutler D., Rogers R.E.L. (2008). Does Fumagillin Control the Recently Detected Invasive Parasite *Nosema ceranae* in Western Honey Bees (*Apis mellifera*)?. J. Invertebr. Pathol..

[43] Gisder S., Genersch E. (2015). Identification of Candidate Agents Active against *N. ceranae* Infection in Honey Bees: Establishment of a Medium Throughput Screening Assay Based on *N. ceranae* Infected Cultured Cells. PLoS ONE.

[44] van den Heever J.P., Thompson T.S., Otto S.J.G., Curtis J.M., Ibrahim A., Pernal S.F. (2016). The Effect of Dicyclohexylamine and Fumagillin on *Nosema ceranae*-Infected Honey Bee (*Apis mellifera*) Mortality in Cage Trial Assays. Apidologie.

[45] Peirson M., Pernal S.F. (2024). A Systematic Review of Fumagillin Field Trials for the Treatment of *Nosema* Disease in Honeybee Colonies. Insects.

[46] Gary N.E., Lorenzen K. (1990). Vacuum Devices for Capturing and Partitioning Commingled Subpopulations of Honey Bees (Hymenoptera: Apidae). Ann. Entomol. Soc. Am..

[47] Traver B.E., Fell R.D. (2011). Prevalence and Infection Intensity of *Nosema* in Honey Bee (*Apis mellifera* L.) Colonies in Virginia. J. Invertebr. Pathol..

[48] Roetschi A., Berthoud H., Kuhn R., Imdorf A. (2008). Infection Rate Based on Quantitative Real-Time PCR of *Melissococcus plutonius*, the Causal Agent of European Foulbrood, in Honeybee Colonies before and after Apiary Sanitation. Apidologie.

[49] Nasr M.E., Thorp R.W., Tyler T.L., Briggs D.L. (1990). Estimating Honey Bee (Hymenoptera: Apidae) Colony Strength by a Simple Method: Measuring Cluster Size. J. Econ. Entomol..

[50] Wood S.C., Kozii I.V., Koziy R.V., Epp T., Simko E. (2018). Comparative Chronic Toxicity of Three Neonicotinoids on New Zealand Packaged Honey Bees. PLoS ONE.

[51] De Jong D., De Andrea Roma D., Goncalves L.S. (1982). A Comparative Analysis of Shaking Solutions for the Detection of *Varroa jacobsoni* on Adult Honeybees. Apidologie.

[52] Lee K.V., Moon R.D., Burkness E.C., Hutchison W.D., Spivak M. (2010). Practical Sampling Plans for *Varroa destructor* (Acari: Varroidae) in *Apis mellifera* (Hymenoptera: Apidae) Colonies and Apiaries. J. Econ. Entomol..

[53] Meikle W.G., Weiss M., Stilwell A.R. (2016). Monitoring Colony Phenology Using Within-Day Variability in Continuous Weight and Temperature of Honey Bee Hives. Apidologie.

[54] Prouty C., Jack C., Sagili R., Ellis J.D. (2023). Evaluating the Efficacy of Common Treatments Used for *Vairimorpha* (*Nosema*) spp. Control. Appl. Sci..

[55] Li J.H., Evans J.D., Li W.F., Zhao Y.Z., DeGrandi-Hoffman G., Huang S.K., Li Z.G., Hamilton M., Chen Y.P. (2017). New Evidence Showing That the Destruction of Gut Bacteria by Antibiotic Treatment Could Increase the Honey Bee’s Vulnerability to *Nosema* Infection. PLoS ONE.

[56] Nozal M.J., Bernal J.L., Martín M.T., Bernal J., Álvaro A., Martín R., Higes M. (2008). Trace Analysis of Fumagillin in Honey by Liquid Chromatography-Diode Array–Electrospray Ionization Mass Spectrometry. J. Chromatogr. A.

[57] Emsen B., De la Mora A., Lacey B., Eccles L., Kelly P.G., Medina-Flores C.A., Petukhova T., Morfin N., Guzman-Novoa E. (2020). Seasonality of *Nosema ceranae* Infections and Their Relationship with Honey Bee Populations, Food Stores, and Survivorship in a North American Region. Vet. Sci..

[58] Copley T.R., Chen H., Giovenazzo P., Houle E., Jabaji S.H. (2012). Prevalence and Seasonality of *Nosema* Species in Québec Honey Bees. Can. Entomol..

[59] Mendoza Y., Diaz-Cetti S., Ramallo G., Santos E., Porrini M., Invernizzi C. (2016). *Nosema ceranae* Winter Control: Study of the Effectiveness of Different Fumagillin Treatments and Consequences on the Strength of Honey Bee (Hymenoptera: Apidae) Colonies. J. Econ. Entomol..

