# Supplementary material for: Fall Treatment with Fumagillin Contributes to an Overwinter Shift in Vairimorpha Species Prevalence in Honey Bee Colonies in Western Canada
Source: Life (Basel). 2024 Mar 12;14(3):373. doi: 10.3390/life14030373 (PMC10971274; doi:10.3390/life14030373)
Supplement: Supplementary file 1 [file life-14-00373-s001.zip › S1_statistics spores.pdf]

Table S1 A, B, C. Friedman's repeated measures ANOVA statistical analysis for spore loads on different time points in control and fumagillin treatment group. (A) Mixed effects model, (B) pairwise comparison between treatments grouped by time points, (C) pairwise comparison between time points grouped by treatment.

A

Effect	DFn	DFd	F	p	p adjust	significant
**Treatment**	1	208	7.854	0.0063	0.036	*
**Time**	3	208	41.114	0.0000	0.372	*
**Treatment:Time**	3	208	2.247	0.084	0.031	

B

Time	Group 1	Group 2	N1	N2	Statistics	Df	p adjust	significant
**21-Sep**	control	fumagillin	27	27	0.794	26	0.434	ns
**8-Oct**	control	fumagillin	27	27	3.51	26	0.002	**
**23-Mar**	control	fumagillin	27	27	1.53	26	0.139	ns
**19-May**	control	fumagillin	27	27	1.99	26	0.057	ns

C

Treatment	Group 1	Group 2	N1	N2	statistics	Df	p	p adjust	significant
**Control**	19-May	21-Sep	27	27	5.103639	26	0.0000	0.0002	***
**Control**	19-May	23-Mar	27	27	4.642979	26	0.0001	0.0005	***
**Control**	19-May	8-Oct	27	27	5.152633	26	0.0000	0.0001	***
**Control**	21-Sep	23-Mar	27	27	-2.53181	26	0.0180	0.1060	ns
**Control**	21-Sep	8-Oct	27	27	0.059268	26	0.9530	1.0000	ns
**Control**	23-Mar	8-Oct	27	27	2.655083	26	0.0130	0.0800	ns
**Fumagillin**	19-May	21-Sep	27	27	8.711635	26	0.0000	0.0000	****
**Fumagillin**	19-May	23-Mar	27	27	7.096466	26	0.0000	0.0000	****
**Fumagillin**	19-May	8-Oct	27	27	9.442285	26	0.0000	0.0000	****
**Fumagillin**	21-Sep	23-Mar	27	27	-2.53548	26	0.0180	0.1060	ns
**Fumagillin**	21-Sep	8-Oct	27	27	4.039412	26	0.0004	0.0030	**
**Fumagillin**	23-Mar	8-Oct	27	27	4.24913	26	0.0002	0.0010	**
