# Supplementary material for: Fall Treatment with Fumagillin Contributes to an Overwinter Shift in Vairimorpha Species Prevalence in Honey Bee Colonies in Western Canada
Source: Life (Basel). 2024 Mar 12;14(3):373. doi: 10.3390/life14030373 (PMC10971274; doi:10.3390/life14030373)
Supplement: Supplementary file 1 [file life-14-00373-s001.zip › S2_statistics species.pdf]

Table S2 A, B, C. Friedman's repeated measures ANOVA statistical analysis for *V. ceranae* prevalence (%) on different time points in control and fumagillin treatment group. (A) Mixed effects model, (B) pairwise comparison between treatments grouped by time points, (C) pairwise comparison between time points grouped by treatment.

A

Effect	DFn	DFd	F	p	p adjust	significant
**Treatment**	1	208	20.403	0.00001	0.089	*
**Time**	3	208	6.908	0.002	0.091	*
**Treatment:Time**	3	208	1.230	0.300	0.017	*

B

Time	Group 1	Group 2	N1	N2	Statistics	Df	p adjust	significant
**21-Sep**	Control	Fumagillin	27	27	0.874	26	0.39	ns
**8-Oct**	Control	Fumagillin	27	27	-1.67	26	0.107	ns
**23-Mar**	Control	Fumagillin	27	27	-2.79	26	0.01	**
**19-May**	Control	Fumagillin	25	25	-4.75	27	0.00006	****

C

Treatment	Group 1	Group 2	N1	N2	Statistics	Df	p	p adjust	significant
**Control**	19-May	21-Sep	27	27	-5.10059	26	0.0000	0.000155	***
**Control**	19-May	23-Mar	27	27	-2.5684	26	0.0160	0.098	ns
**Control**	19-May	8-Oct	27	27	-4.57549	26	0.0001	0.000618	***
**Control**	21-Sep	23-Mar	27	27	1.426178	26	0.1660	0.996	ns
**Control**	21-Sep	8-Oct	27	27	0.13193	26	0.8960	1	ns
**Control**	23-Mar	8-Oct	27	27	-1.36204	26	0.1850	1	ns
**Fumagillin**	19-May	21-Sep	27	27	-1.56507	26	0.1300	0.78	ns
**Fumagillin**	19-May	23-Mar	27	27	-2.56818	26	0.0160	0.098	ns
**Fumagillin**	19-May	8-Oct	27	27	-2.24563	26	0.0330	0.2	ns
**Fumagillin**	21-Sep	23-Mar	27	27	-0.61484	26	0.5440	1	ns
**Fumagillin**	21-Sep	8-Oct	27	27	-0.5995	26	0.5540	1	ns
**Fumagillin**	23-Mar	8-Oct	27	27	0.058692	26	0.9540	1	ns
